# Multilayer Graphtriyne Membranes for Separation and Storage of CO_2_: Molecular Dynamics Simulations of Post-Combustion Model Mixtures

**DOI:** 10.3390/molecules27185958

**Published:** 2022-09-13

**Authors:** Yusuf Bramastya Apriliyanto, Noelia Faginas-Lago, Stefano Evangelisti, Massimiliano Bartolomei, Thierry Leininger, Fernando Pirani, Leonardo Pacifici, Andrea Lombardi

**Affiliations:** 1 Department of Chemistry, The Republic of Indonesia Defense University, Kampus Unhan Komplek IPSC Sentul, 16810 Bogor, Indonesia; 2 Department of Chemistry, Biology and Biotechnology, University of Perugia, & UdR INSTM di Perugia, Via Elce di Sotto 8, 06123 Perugia, Italy; 3 Consortium for Computational and Materials Sciences (CMS)^2^, Via Elce di Sotto, 8, 06123 Perugia, Italy; 4 Laboratoire de Chimie et Physique Quantiques, IRSAMC, Université de Toulouse III-Paul Sabatier, 118 Route de Narbonne, CEDEX 09, 31062 Toulouse, France; 5 Instituto de Física Fundamental, Consejo Superior de Investigaciones Científicas (IFF-CSIC), Serrano 123, 28006 Madrid, Spain

**Keywords:** carbon dioxide, graphyne, molecular dynamics, CO_2_ capture

## Abstract

The ability to remove carbon dioxide from gaseous mixtures is a necessary step toward the reduction of greenhouse gas emissions. As a contribution to this field of research, we performed a molecular dynamics study assessing the separation and adsorption properties of multi-layered graphtriyne membranes on gaseous mixtures of CO_2_, N_2_, and H_2_O. These mixtures closely resemble post-combustion gaseous products and are, therefore, suitable prototypes with which to model possible technological applications in the field of CO_2_ removal methodologies. The molecular dynamics simulations rely on a fairly accurate description of involved force fields, providing reliable predictions of selectivity and adsorption coefficients. The characterization of the interplay between molecules and membrane structure also permitted us to elucidate the adsorption and crossing processes at an atomistic level of detail. The work is intended as a continuation and a strong enhancement of the modeling research and characterization of such materials as molecular sieves for CO_2_ storage and removal.

## 1. Introduction

The persistent growth of greenhouse gas emissions, the debate about the role of anthropic activities in connection with climate change [1], and the global warming phenomenon [2] have stimulated the search for “clean” technologies that can achieve carbon dioxide removal from gas mixtures such as the flue gases generated after combustion. Selective adsorption using porous materials is a promising way to capture CO_2_, which is mostly generated by fossil fuel combustion, in order to mitigate the greenhouse effects associated with its excessive concentration in the atmosphere [3,4,5]. This method is favored in terms of its simplicity and lower implementation costs, in comparison with the more traditional aqueous chemical absorption [6].

A range of porous materials, such as nano-porous carbons [7,8,9,10], zeolites, the zeolitic imidazolate framework (ZIFs) [11,12], metal-organic frameworks (MOFs) [13,14,15,16], porous polymer networks (PPNs) or covalent organic frameworks/polymers (COFs/COPs) [17,18,19,20], and slurries made of solid adsorbents in a liquid absorbent [21] have been proposed for CO_2_ capture over the past few years. An alternative to porous adsorbing materials is represented by nano-porous membranes that offer a combination of surface adsorption and the action of pores as a molecular sieve to separate CO_2_ from other gases [22,23]. In this category, recently, carbon-based membranes emerged as potentially useful materials because of some remarkable properties, e.g., they are hydrophobic, chemically inert, and thermally stable, with a practical implementation that is economically viable for post-combustion CO_2_ capture and separation [24,25,26,27,28]. MOFs and polymers, for instance, although exhibiting good selectivity and permeability, are susceptible to heat and water vapor, which is a characteristic of post-combustion flue gases.

In practice, it is a very difficult or even impossible task to experimentally synthesize, characterize, and evaluate the performance in terms of the CO_2_ capture and separation of all possible materials. Up to this point in the process, computer modeling and simulations play an important role in material design and development, prior to the experimental stage [29,30]. Due to the variety of interactions between molecules and materials, generic force fields like AMBER [31] and UFF [32] often poorly model the particular system. Thus, parts of the potential energy function must be developed or refined on purpose, using the available theoretical and experimental data. Recently, various force fields specific to graphene and its derivatives have been developed [33,34], as well as those for MOFs [35,36,37], zeolites [38], and other polymeric materials [39], to identify molecular interactions providing realistic predictions of relevant adsorption dynamics and the transport properties of gas under consideration.

However, intermolecular interaction parameterization is a delicate task and the accurate formulation of force fields is an indispensable work: they must be reliable in terms of the full space of the relative configurations of involved partners and must be expressed in a proper analytical form, to permit extensive molecular dynamics (MD) simulations under a variety of conditions of interest. The complete achievement of this knowledge is a very critical question, one that is also difficult to answer for relatively simple systems. This has been one important target of our recent research activity. We have found that the strength, radial, and angular dependencies of the basic interaction components are definable by semi-empirical and empirical functions, the parameters of which relate to the fundamental chemical-physical properties of the interacting partners. Under these conditions, such functions become scaling laws, the involved parameters assume a defined transferability degree, and both gas-gas and gas-layer intermolecular interactions become representable in an internally consistent way.

Permeability and selectivity are the two main aspects by which to determine whether a membrane can be effective for gas separation. It is already well known that permeability is inversely proportional to the thickness of the membrane. Therefore, a single-atom-thick planar membrane may have great potential for gas separation if tailored to be selective for a given molecule [40,41]. The γ-graphynes are single atomic layers belonging to the class of carbon allotropes wherein hexagonal carbon rings are connected by carbon chains containing a variable number of C-C acetylenic bonds. The γ-graphynes exhibit similar properties to graphene, but the pores are uniformly distributed and have adjustable dimensions [42]. Moreover, graphynes have lower dispersion forces that minimize aggregate formation among the layers and molecules. The synthesis and characterization techniques of graphynes have actively been developed over the last few years [43,44,45,46,47,48]. In our previous works [49,50], force fields related to gas adsorption on γ-graphynes have also been developed and tested using accurate ab initio calculations. Therefore, in this work, we will perform extended molecular dynamics (MD) simulations on a wide range of conditions that are typical of post-combustion gaseous mixtures containing CO_2_/N_2_/H_2_O, in order to characterize the separation properties of multi-layer graphtriyne membranes, a γ-graphyne characterized with three consecutive C-C acetylenic bonds.

## 2. Methods

The classical MD simulations of graphtriyne and gas mixtures were performed by enforcing periodic boundary conditions with a simulation box with the dimensions 72.210 Å × 62.523 Å × 280.0 Å. The box contained graphtriyne membrane(s) with the dimensions 72.210 Å × 62.523 Å, placed at the midway point perpendicularly to the z-direction. Three different types of arrangements, with 1, 2, and 3 membrane layers at four different temperatures (333, 353, 373, and 400 K), were the subject of simulations with CO_2_/N_2_/H_2_O gaseous mixtures, with equal concentrations of CO_2,_ N_2_, and H_2_O. The structures of the graphtriyne membranes were taken from Ref. [49], where they had been optimized using periodic DFT calculations (the structural details are reported in the Supplementary Information, in Figure S1).

**Figure 1:**
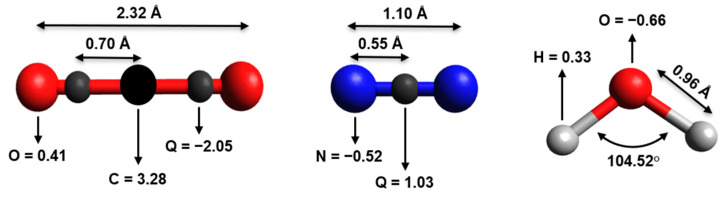
A schematic view of carbon dioxide, nitrogen, and water molecules, with the atomic charges (a.u.), angles, and bond lengths adopted. Charge-sites (Q) are represented by the smaller gray spheres.

As previously reported by the authors of [50], the intermolecular potential has been defined as a combination of electrostatic and non-electrostatic components. The first component is represented by the sum of Coulomb interactions between the partial point charges located on each molecular frame, the anisotropic distribution of which accounts for the permanent electric multipole of each partner. In particular, for H_2_O, the representation reported by the authors of [51] was adopted, with a charge distribution correctly reproducing the dipole moment of water in the gas phase (1.85 D) [52], while a three-charge-site N_2_ model [53] and a five-charge-site CO_2_ model [54] were used (see Figure 1).

The second (non-electrostatic) component of the intermolecular potential, acting between pairs of gas molecules and between the gas molecules and the membranes, is determined by the balance of size repulsion with dispersion and induction attraction. As previously, it has been described by pair-wise additive contributions, arising from the different interaction centers distributed again on each molecular frame, and formulated using the improved Lennard-Jones (ILJ) potential function [55]. The ILJ expression, adopted in place of the much simpler Lennard-Jones function (usual in generic force fields) describes non-electrostatic intermolecular interactions in a far more accurate way [56,57,58]. All the ILJ parameters used in this work, predicted from the polarizability component assigned at each interaction center, were tested and fine-tuned using the available experimental findings, exploiting the comparison of interaction energies with the results of high-level ab initio calculations [49,50]. A cut-off distance for the ILJ and electrostatic interactions was set to be equal to 15 Å. Given the periodic boundary conditions, the smoothed particle mesh Ewald method, as implemented in the DL_POLY software (see below), was applied to accurately account for long-range electrostatic interactions [59].

**Figure 2:**
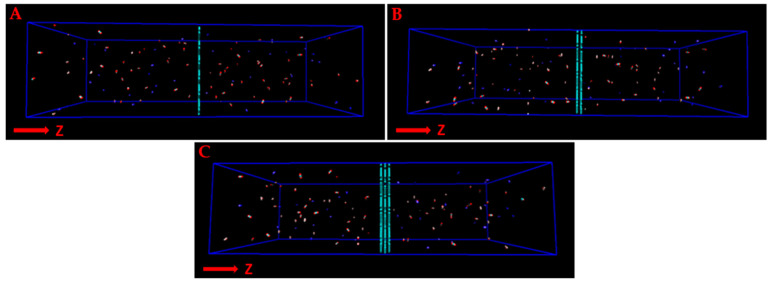
The simulation box, filled with a gaseous mixture of CO_2_, N_2_, and H_2_O. Single (**A**), double (**B**), and triple (**C**) layers of graphtriyne are shown.

All MD simulations were performed using the DL_POLY molecular dynamics program [60] in the canonical (NVT) ensemble, employing the Nosé–Hoover thermostat with periodic boundary conditions in all directions. Each simulation was carried out for a period of 5.5 ns, after a 0.5 ns equilibration period, with a fixed time step of 1 fs; the trajectory data for the statistics were collected every 2 ps. Seven different amounts of gas have been loaded into the simulation box for every system to characterize the influence of pressure on the observable objective of the present investigation. The resulting gas pressure was computed using the Peng–Robinson equation of state [61]. In order to mimic post-combustion conditions, the initial pressure values were chosen to be lower than 5.5 atm (the details are reported in the Supplementary Information, in Tables S1–S4). At the beginning of the simulations, equal amounts of the gas molecules were randomly distributed into the two regions of the box, in such a way that there was no pressure gradient inside the box (Figure 2). The membranes were considered a frozen framework and the gas molecules were treated as rigid bodies. The gas molecules could cross the membrane multiple times in both directions of the *z*-axis during the simulation. The number of permeation events was then monitored, along with the z-density and radial distribution function profiles. All graphical representations of the molecular trajectories were processed using the VMD package [62].

## *3.* Results and Discussion

### 3.1. Gas Permeability

**Figure 3:**
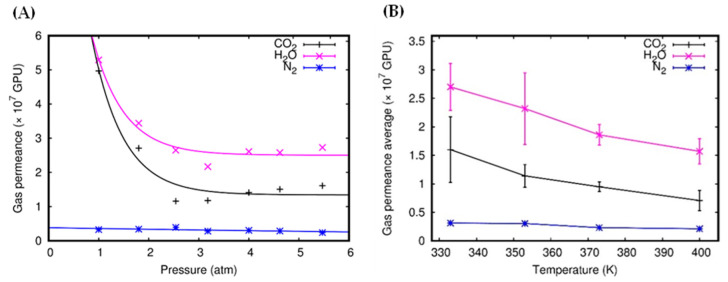
Gas permeance of the single-layer system at 333 K (**A**) and gas permeance average of the single-layer system as a function of temperature (**B**).

The simulations were considered to be initialized after the equilibration steps when the permeation events that occurred were monitored and counted. The numbers of permeation events were then plotted against the simulation time. By definition, the slope of such plots represents an estimation of the gas permeation rate, measured in units of molecules ps^−1^. Using these data, the gas permeance was then calculated by dividing the permeation rate by the corresponding pressure and by the area of the membrane. The gas permeances of the single layer are reported in Figure 3, using the gas permeance unit (GPU), where 1 GPU is equal to 3.35 × 10^−10^ mol m^−2^ s^−1^ Pa^−1^. Figure 3A shows that the N_2_ permeances are not affected by the pressures. Meanwhile, the CO_2_ and H_2_O permeances are higher at low pressure and show a relatively flat trend at increasing pressures. However, for all gas molecules, the permeances do not vary much at pressures higher than 2 atm.

**Figure 4:**
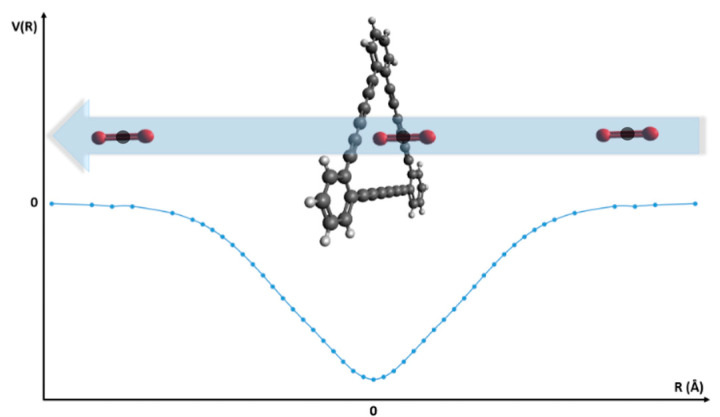
An illustration of the permeation process of CO_2_, displayed through a pore unit of the graphtriyne layer (adopted from the potential energy curve reported in Ref. [50]).

However, upon closer scrutiny, the data in Figure 3A, although shown in the limited pressure range considered, seem to suggest a minimum level of permeance, followed by a rebound profile. Such a trend, in principle, is to be expected, due to the increase in the frequency of collisions that is typical of increasing pressures. However, more simulations covering a wider pressure range would be needed to confirm and reproduce this behavior. For the range where the permeances are weakly dependent on pressure, we calculated the average of the gas permeance for each gas and plotted it as a function of temperature (Figure 3B). The plot shows that the average gas permeance decreases as the temperature increases for all the molecules. This behavior is likely to be a consequence of the increased kinetic energy of the molecules; by possessing higher kinetic energies (velocities), molecules more efficiently escape the attraction forces of the membrane. Therefore, the high temperature decreases the gas permeance by contrasting the attraction effects that steer the molecules toward the membrane. The low average permeance of N_2_ in the entire range of temperatures considered in the simulations indicates that the attractive forces between the membrane and N_2_ are considerably weaker, compared to those experienced by CO_2_ and H_2_O. This fact is reflected in the potential energy profiles reported by Bartolomei and co-workers [27,49,50], describing the membrane-molecule interactions, showing that N_2_ has the smallest well depth, followed by H_2_O and CO_2_. In spite of having the deepest potential energy well, CO_2_ demonstrates permeance values lower than those of H_2_O. To explain such apparent illogicality requires more insight into the permeation process, schematized in Figure 4.

First, the molecules located near the membrane are affected by the attraction forces and move toward the pores. In this step, the CO_2_ molecules show a propensity toward more efficient adsorption by the membrane, due to its strong attraction forces. Then, a gas molecule can reach the center of the pore (z=0), a stable configuration where the potential energy is minimal. If there is enough kinetic energy to overcome the attraction forces, the molecule will cross the membrane and fly to the other side. On the other hand, if the molecule does not have enough energy, it will remain adsorbed by the membrane until it acquires extra energy (collisions with other molecules) or assumes an optimal orientation for crossing. Due to the deeper potential well, CO_2_ molecules need greater kinetic energy than H_2_O to overcome the attraction forces and to successfully cross the membrane. Moreover, in the case of CO_2_, the permeation is stereoselective with respect to the molecular orientation. The penetration process encounters energy barriers when CO_2_ molecules approach the membrane in parallel configurations (see Figure 1 in Ref. [50] for the energy profiles corresponding to the perpendicular and parallel approach, and for the related discussions). This qualitatively explains why CO_2_ molecules find it more difficult to pass through the membrane, even though the numbers of CO_2_ molecules attracted and adsorbed by the membrane are larger than those of H_2_O (this can be seen in the z-density profiles, which will be discussed later). Therefore, as can be seen in Figure 3 for the single-layer systems, H_2_O permeances are the highest ones.

**Figure 5:**
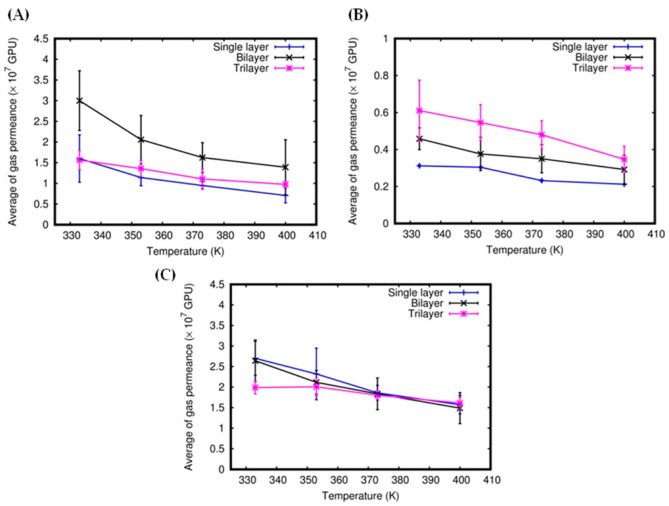
Average of gas permeance as a function of temperature: CO_2_ (**A**), N_2_ (**B**), and H_2_O (**C**).

The gas permeances of the multilayer graphtriyne can be described using the data presented in Figure 5. We can see that the N_2_ average permeances increase as the number of layers increases (Figure 5B). This behavior is consistent with the increasingly stronger attractive forces probed by N_2_ that the graphtryine is subjected to, passing from bi- to trilayers, as can be seen from the interaction energies of N_2_ with the multilayers reported in Ref. [49]. However, CO_2_ shows different behavior since the highest permeances, which are achieved in the bilayer system, decrease in the trilayer membrane (Figure 5A). This is the consequence of the efficient adsorption of CO_2_ in the interlayer regions between the three graphtriyne sheets (this aspect will be discussed later). The presence of strongly adsorbed molecules prevents other CO_2_ molecules from crossing the membranes.

Although the trend of permeances is consistent with the ones obtained in Ref. [50], the values reported here are lower (ranging from 0.6 to 3.0 × 10^7^ GPU and from 0.3 to 0.6 × 10^7^ GPU for CO_2_ and N_2_, respectively). Lower overall values of the gas permeances are a natural consequence of the presence of three different species comprising CO_2_/N_2_/H_2_O, competing to interact with the membrane in the gaseous mixtures (while the mixtures in Ref. [50] were binary) and by the higher temperatures characterizing the present simulations. In the case of H_2_O (Figure 5C), the average gas permeances do not change greatly according to the number of layers. Moreover, H_2_O permeances are significantly higher than those of CO_2_ and N_2_ for the single and trilayer systems (much less at low temperatures (see Figure 5)), with the noticeable exception of the bilayer system. Figure 5 also shows that the gas permeances for all the molecules decrease with increasing temperature for single-layer, bilayer, and trilayer systems (see also Figure 3B).

**Figure 6:**
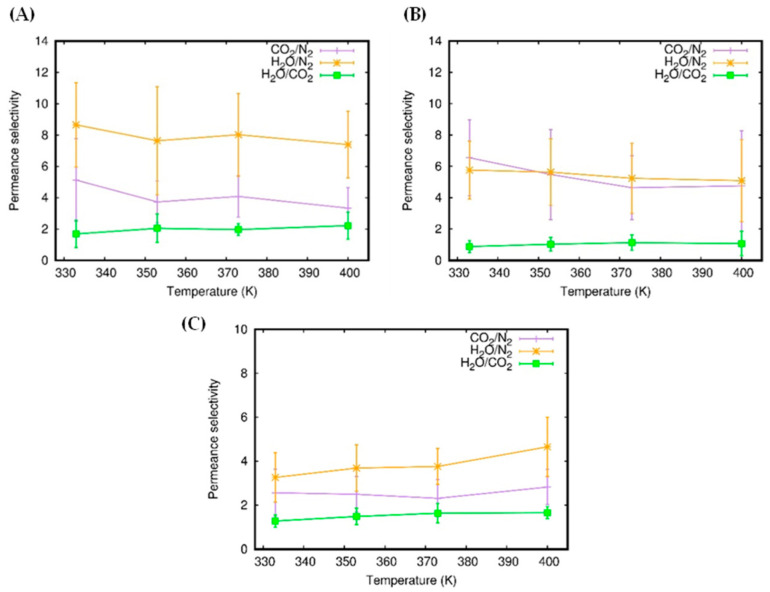
Permeance selectivity for single-layer (**A**), bilayer (**B**), and trilayer (**C**) systems.

By comparison of the gas average permeances, we calculated the permeance selectivity values (obtained, for a pair of molecules, as the ratio of the individual gas permeances) and plotted them as a function of temperature for all types of membrane systems and pairs of molecules. The results are reported in Figure 6. It can be seen that the permeance selectivity is affected by temperature, so that we can assume such a dependence, with the caveat that with the variations being lower than the standard error, the effect could be weak or poorly significant in the observed temperature range. The permeance selectivity of the H_2_O/N_2_ pair is the highest one in both single-layer and trilayer systems, while it essentially overlaps that of CO_2_/N_2_ in the bilayer system. Interestingly, H_2_O/CO_2_ selectivity is ~1 or slightly higher for both bi- and trilayer systems, indicating that such membranes are not selective for CO_2_-H_2_O separation. Figure 6 also shows that, in general, the trilayer system (C) has permeance selectivity values that are lower than for the single (A) and bilayer (B) systems. These are related to efficient molecular adsorption in the interlayer regions of the trilayer system, which can lower gas permeance (this aspect will be discussed in the next section).

Although the CO_2_/N_2_ permeance selectivity values obtained here are much lower than those reported for nanoporous graphene at 300 K by Liu et al. [23] (about 100, with CO_2_ permeance = 2.8 × 10^5^ GPU) and by Schrier [17] (about 60, with CO_2_ permeance = 3 × 10^5^ GPU) for porous graphene-E-stilbene-1 (PG-ES1) at 325 K; the CO_2_ permeances for bilayer graphtriyne (ranging from 0.6 to 3.0 × 10^7^ GPU) are two orders of magnitude larger. Moreover, the CO_2_/N_2_ permeance selectivity values for the bilayer system are comparable to and higher than the value (5.4) reported in our previous work for CO_2_/N_2_ binary mixtures at 300 K [50]. The CO_2_/N_2_ permeance selectivity values are also comparable with those reported by Wu and co-workers [24] for fluorine-modified nanoporous graphene at 300 K (ranging from 4 to 11). This indicates that the bilayer graphtriyne membrane could represent an efficient molecular sieve to be used in an initial separation step for CO_2_ post-combustion separation; the flue gas is mainly composed of molecular nitrogen.

### 3.2. Gas Adsorption

**Figure 7:**
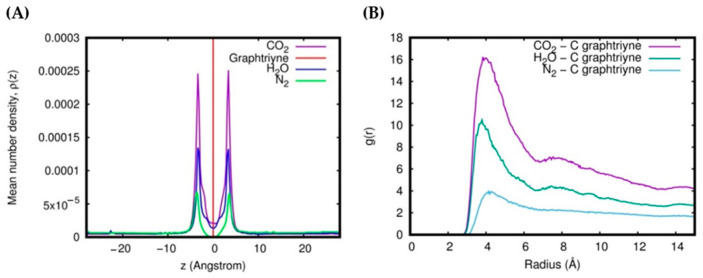
z-Density profile (**A**) and radial distribution function (**B**) of the single-layer system at 1 atm and 353 K.

The adsorption process can be characterized by the mean number densities of gas molecules along the z-axis (the direction perpendicular to the graphtriyne membrane). For the single-layer system, these z-density profiles, ρ(z), tend to peak around a distance of 3.4 Å from the surface in all gas molecules (Figure 7A). The strength of the attraction exerted by the membrane affects the height of the z-density profile peaks; accordingly, CO_2_ shows the highest peaks. We can also see from the radial distribution functions g(r) (Figure 7B) that CO_2_ has the highest probability to be found near the carbon atoms of the graphtriyne (around 4 Å). The strong peaks of CO_2_ in the z-density and the radial distribution function are a consequence of the deep and wide potential well that characterizes the CO_2_–membrane interaction potential profile, resulting in a strong long-range attraction (see Figure 4 and Refs. [49,50]). As already discussed in the previous section, the permeation events are closely related to the adsorption of gas molecules over the surfaces of the membranes. The more the molecules are adsorbed, the higher their permeance. However, the stereodynamic requirements of CO_2_ for the membrane-crossing process (which is reported and exhaustively discussed in our previous work [50]) lead to permeances lower than those of H_2_O.

The gas uptake can be estimated by integrating the area under the peaks of the z-density profiles. The interval of integration along the *z*-direction is the adsorption region located within ± 6.9 Å with respect to the membrane position of the single-layer system. Notice that the z-density does not peak at the center of the pore (z=0), where, according to the potential profiles (see Figure 4), we see the energy minimum. This feature of z-density has already been discussed in our previous report [50] and has been interpreted, in terms of the oscillatory motion of the molecule in a physisorption state, as being due to the longer residence times at turning points and, in the case of CO_2_, are also enhanced by the stereodynamics of the crossing process [49].

**Figure 8:**
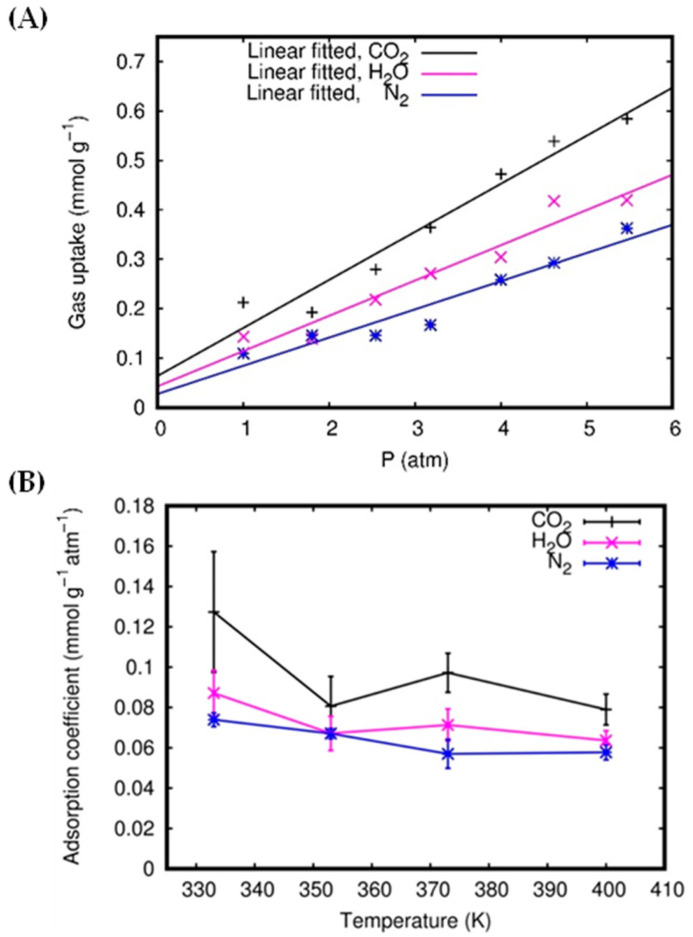
Single-layer system: the adsorption isotherms at 373 K (**A**) and adsorption coefficients as a function of temperature (**B**).

The gas uptakes for all applied pressures are reported in Figure 8A in the form of adsorption isotherms. We can see that the gas uptake is linearly related to the initial pressures and increases with the increasing values of pressure. Therefore, using linear regression, we can estimate the adsorption coefficients as the slopes of the linear functions fitting the adsorption isotherms. The adsorption coefficients for all gas molecules in the single-layer system are reported in Figure 8B, as a function of the temperature. It can be seen that CO_2_ has the highest gas uptakes and adsorption coefficients among the gas molecules. On the other hand, despite the fact that H_2_O has the highest permeance, its gas uptakes and adsorption coefficients are lower, again indicating weaker attraction forces than those acting for CO_2_. The weak attraction of N_2_ by the membrane is manifested by the fact that N_2_ has the lowest adsorption coefficient (about 0.06 to 0.07 mmol g^−1^ atm^−1^). Figure 8B shows that the adsorption coefficients decrease for increasing temperature. This trend appears to fluctuate at 350 K; this still leaves room for a different trend than the expected linear one. However, a much longer simulation and a wider temperature range are needed to obtain a clear view. Obviously, with higher temperatures, the gas molecules have a higher tendency to escape; thus, the physisorption is expected to be less effective.

**Figure 9:**
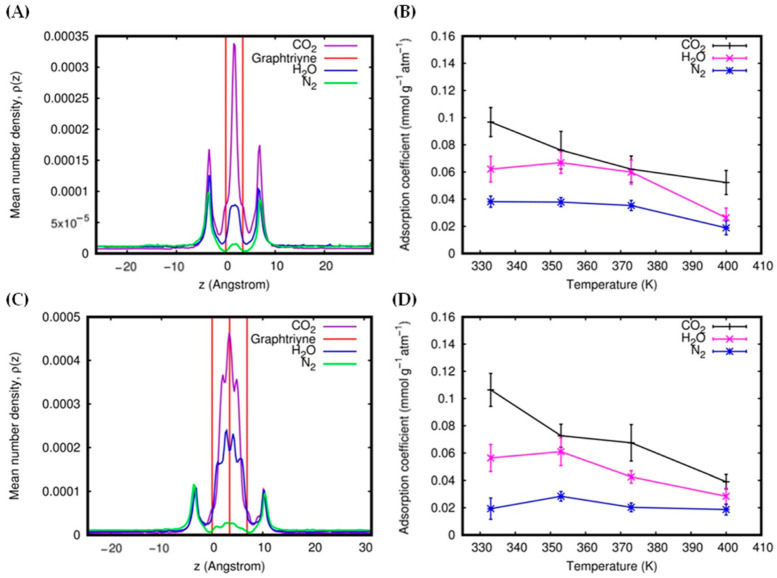
*z*-Density profiles at 1.8 atm and 353 K for bilayer (**A**) and trilayer (**C**) membranes. Adsorption coefficient as a function of temperature for bilayer (**B**) and trilayer (**D**) membranes.

Similar trends are observed in the case of bilayer and trilayer systems. CO_2_ shows the highest gas uptake and adsorption coefficients (Figure 9B,D), and the adsorption coefficients also depend on the temperature: higher temperatures provide lower adsorption coefficients. Unlike the single-layer system, in the bilayer and trilayer systems, we find z-density peaks in the interlayer regions (Figure 9A,C). Therefore, it is expected that the bilayer and trilayer membranes should be more efficient in adsorbing molecules due to their interlayer pores (as reported in the Supplementary Information, Tables S11–S14). However, adsorbed molecules can, in some conditions, also contribute to diminishing the gas permeance by saturating the pores and preventing other molecules from crossing the membrane. This phenomenon can be observed in the case of CO_2_ with trilayer systems, due to the strong attraction and high uptake. Indeed, we can see from Figure 5A that the CO_2_ permeance values significantly increase when passing from single-layer to bilayer systems, then collapsing to values typical of single layers when passing from bilayer to trilayer systems. This phenomenon can then accumulate into a decrease in the value of the permeance selectivity of the trilayer system, relative to other systems, as shown in Figure 6C.

**Figure 10:**
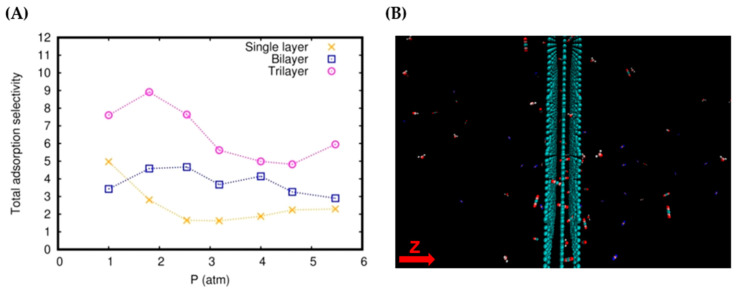
Total adsorption selectivity of CO_2_/N_2_ at 333 K (**A**) and a snapshot of the configurations for the trilayer system at 4.62 atm and 353 K (**B**).

The interlayer region is relatively selective to CO_2_ molecules (see Figure 9A,C); for instance, we obtained interlayer adsorption selectivity of CO_2_/N_2_ = 20.23, CO_2_/H_2_O = 1.85 for the bilayer system at 333 K and 4.00 atm, with CO_2_/N_2_ = 42.45, CO_2_/H_2_O = 2.38 for the trilayer system at 400 K and 2.54 atm (see the Supplementary Information, Table S18). The interlayer adsorption refers to the adsorption values in the interlayer region, i.e., between the two outermost red lines of the z-density profiles (see Figure 9A,C). The high interlayer selectivity is an additional feature of multilayer graphtriyne membranes. However, for most practical applications, it is better to express the selectivity in terms of the total adsorption selectivity. This is because the available experimental and theoretical data reported in the literature often only report the total adsorptions (in pores + surface adsorptions). The total adsorption refers to the sum of the adsorption values in the interlayer region and in the outer (adsorption) region, within ±6.9 Å from the membrane position. The interlayer and total adsorption selectivity relative to two molecules, *A* and *B*. $S_{ads}^{A/B}$, was calculated according to the formula:

$$S_{ads}^{A/B}=\frac{n_{A\;\left(ads\right)}}{n_{A\;\left(free\right)}}\times \frac{n_{B\;\left(free\right)}}{n_{B\;\left(ads\right)}}$$

where $\;\;n_{A\;\left(ads\right)}$ and $n_{B\;\left(ads\right)}$ are the numbers of adsorbed molecules *A* and *B*, respectively, while $n_{A\;\left(free\right)}$ and $n_{B\;\left(free\right)}$ are the numbers of free molecules of *A* and *B*, respectively [63]. The total adsorption selectivity values for all the systems are reported in Table 1, while some of the data are plotted in Figure 10A. Figure 10 and Table 1 show that trilayer graphtriyne exhibits relatively high CO_2_/N_2_ total adsorption selectivity, especially at low pressure and temperature. As an example, at a temperature of 353 K, the CO_2_/N_2_ total adsorption selectivity ranges from 4.10 to 19.45, while the CO_2_/H_2_O total adsorption selectivity ranges from 1.59 to 11.08, depending on the applied initial pressure.

These CO_2_/N_2_ total adsorption selectivity values are comparable to those of some porous materials for CO_2_ capture applications, such as covalent organic polymers (8.4–13.7 at 1.01 bar, 298 K) [18], metal-organic frameworks (5.0–40.0 at 20 bar, 298 K) [14], and functionalized graphitic slit pores (5.0–20.0 at 20 bar, 298 K) [63]. We can also visually verify the selectivity by looking at the snapshot of a configuration sampled from a typical simulation run, as shown in Figure 10B, where the CO_2_ molecules that are adsorbed are predominant. In general, the total adsorption selectivity of all the gas pairs shows a decreasing trend as the pressure and temperature increase (Table 1 and Figure 10A).

As a matter of fact, the CO_2_/N_2_ total selectivity values reported in Table 1 are higher than those given in our previous work [50]. For instance, here, we obtained the values of the CO_2_/N_2_ total adsorption selectivity for the trilayer system, ranging from 4.8 to 8.9 (trinary gaseous mixture at 333 K), while in our previous work, we reported values ranging from 4.0 to 8.0 (binary gaseous mixture at 300 K). However, the gas uptake values and the corresponding adsorption coefficients are relatively lower than those obtained in the previous work. It is obvious that this difference comes from the different kinds of gaseous mixtures and conditions applied in the simulations. Nevertheless, both reports are in agreement, predicting that the trilayer membrane exhibits a high selectivity for CO_2_ capture. Therefore, when presenting all the advantages of carbon-based materials, multilayer graphtriyne membranes are promising alternatives for post-combustion CO_2_ capture and separation. In particular, the bilayer graphtriyne membrane, with its permeability and permeance selectivity, exhibits good performance as an initial molecular sieve candidate for post-combustion CO_2_ separation, whereas trilayer graphtriyne, with its high gas uptake and adsorption selectivity, is comparable and competitive with other carbon-based adsorbing materials for post-combustion CO_2_ capture.

## 4. Conclusions

In this work, we performed extensive molecular dynamics simulations to assess the suitability of multi-layer graphtriyne membranes for CO_2_ capture and separation. To improve the accuracy of the results, we adopted a proper formulation for the involved force fields, which represent, in an internally consistent way, the fundamental interaction components for both molecule-molecule and molecule-layer (surface) systems. Comprehensive sets of values for the permeance, uptake, and selectivity coefficients have been obtained, which also provide a dynamic and stereodynamic interpretation of the observed data in an interval of temperatures and pressures of interest for many applications. Remarkably, this data set can justifiably be considered predictive in terms of the care taken in the description of the atomistic molecular dynamics and intermolecular interactions, as derived in previous works. Having uniformly distributed and tunable pores, with all the advantages of carbon-based materials, multilayer graphtriyne membranes are promising candidates for the separation and storage of CO_2_ from post-combustion flue gases composed of CO_2_, N_2_ and H_2_O gaseous mixtures. The bilayer graphtriyne membrane represents a good alternative as a molecular sieve for CO_2_ separation, while the trilayer graphtriyne membrane is promising for post-combustion CO_2_ storage and is competitive compared to other carbon-based adsorbing materials. Further developments include the extension of the approach to new carbon-based materials and molecules and the construction of a general force field for accurate simulations of gas-membrane systems.

## Figures and Tables

**Table 1:** Total adsorption selectivity of the CO_2_/N_2_/H_2_O gaseous mixture.

Temperature (K)	Gas	System	Total Adsorption Selectivity						
			1.00 atm	1.80 atm	2.54 atm	3.18 atm	4.00 atm	4.62 atm	5.47 atm
333	CO_2_/N_2_	Single layer	4.97	2.81	1.65	1.63	1.88	2.24	2.30
		Bilayer	3.43	4.58	4.67	3.67	4.14	3.26	2.90
		Trilayer	7.61	8.92	7.64	5.62	4.99	4.82	5.95
	H_2_O/N_2_	Single layer	2.44	1.80	1.30	1.10	1.38	1.48	1.39
		Bilayer	3.00	2.84	2.40	1.65	2.10	1.86	2.12
		Trilayer	3.73	3.07	3.74	2.38	2.65	2.23	2.91
	CO_2_/H_2_O	Single layer	2.04	1.56	1.28	1.48	1.36	1.52	1.66
		Bilayer	1.14	1.62	1.95	2.22	1.97	1.75	1.37
		Trilayer	2.04	2.91	2.04	2.36	1.89	2.16	2.05
353	CO_2_/N_2_	Single layer	4.43	1.53	2.43	1.78	1.63	1.73	1.70
		Bilayer	3.19	4.24	3.64	4.19	2.79	2.78	2.60
		Trilayer	19.45	8.85	6.29	4.73	4.60	4.10	4.45
	H_2_O/N_2_	Single layer	2.10	1.40	1.21	1.13	1.11	1.19	1.25
		Bilayer	2.45	1.69	2.08	2.06	1.88	1.93	1.99
		Trilayer	1.76	4.31	3.13	2.20	2.64	2.57	2.53
	CO_2_/H_2_O	Single layer	2.11	1.09	2.01	1.57	1.46	1.45	1.35
		Bilayer	1.30	2.51	1.75	2.03	1.48	1.44	1.31
		Trilayer	11.08	2.05	2.01	2.16	1.74	1.59	1.76
373	CO_2_/N_2_	Single layer	2.30	1.34	2.07	2.37	1.97	1.98	1.70
		Bilayer	5.16	3.04	3.40	2.67	2.62	2.62	2.33
		Trilayer	2.19	2.92	4.12	5.34	4.16	3.78	2.90
	H_2_O/N_2_	Single layer	1.38	0.95	1.56	1.70	1.20	1.48	1.17
		Bilayer	0.81	3.12	2.05	1.42	2.04	1.59	1.84
		Trilayer	2.68	2.99	2.27	2.80	2.34	2.35	2.30
	CO_2_/H_2_O	Single layer	1.67	1.44	1.33	1.40	1.65	1.34	1.45
		Bilayer	6.35	0.97	1.65	1.88	1.28	1.65	1.26
		Trilayer	0.82	0.97	1.81	1.92	1.75	1.61	1.26
400	CO_2_/N_2_	Single layer	2.66	2.23	1.71	1.96	1.83	1.53	1.69
		Bilayer	1.08	3.96	3.12	2.26	2.68	2.54	2.36
		Trilayer	5.26	7.16	7.80	3.66	3.54	2.95	3.53
	H_2_O/N_2_	Single layer	1.05	1.72	0.98	1.39	1.25	1.17	1.13
		Bilayer	1.87	2.00	1.65	1.57	1.75	1.54	1.57
		Trilayer	1.49	3.74	3.60	2.22	2.15	1.63	2.08
	CO_2_/H_2_O	Single layer	2.53	1.30	1.74	1.41	1.46	1.31	1.50
		Bilayer	0.58	1.98	1.89	1.44	1.53	1.66	1.50
		Trilayer	3.53	1.91	2.16	1.65	1.65	1.81	1.70

## Data Availability

The data presented in this study are available in Supplementary Materials.

## Supplementary Materials

The following supporting information can be downloaded at: https://www.mdpi.com/article/10.3390/molecules27185958/s1. Figure S1: Structural details of multilayer graphtriyne; Tables S1–S4: The amount of gas molecules and the corresponding pressure at 333, 353, 373, and 400 K, respectively; Tables S5–S7: Average of gas permeance in single layer, bilayer, and trilayer systems, respectively; Tables S8–S10: Permeance selectivity in single layer, bilayer, and trilayer systems, respectively; Tables S11–S14: Total gas uptake at 333, 353, 373, and 400 K, respectively; Tables S15–S17: Adsorption coefficient in single layer, bilayer, and trilayer systems, respectively; Table S18: Interlayer adsorption selectivity of CO_2_, N_2_, H_2_O gaseous mixture.

## Sample Availability

Samples of the compounds are not available from the authors.
