# Supplementary material for: Multilayer Graphtriyne Membranes for Separation and Storage of CO2: Molecular Dynamics Simulations of Post-Combustion Model Mixtures
Source: Molecules. 2022 Sep 13;27(18):5958. doi: 10.3390/molecules27185958 (PMC9500597; doi:10.3390/molecules27185958)
Supplement: Supplementary file 1 [file molecules-27-05958-s001.zip › molecules-1874698-supplementary.pdf]

Multilayer Graphtriyne Membranes for Separation and Storage of CO_2: Molecular Dynamics Simulations of Post-combustion Model Mixtures

Yusuf Bramastya Apriliyanto [1,*], Noelia Faginas-Lago [2,3,*], Stefano Evangelisti [4], Massimiliano Bartolomei [5], Thierry Leininger [4], Fernando Pirani [2], Leonardo Pacifici [2] and Andrea Lombardi [2,3,*]

1 Department of Chemistry, The Republic of Indonesia Defense University, Kampus Unhan Komplek IPSC Sentul, 16810 Bogor, Indonesia
2 Department of Chemistry, Biology and Biotechnology, University of Perugia, & UdR INSTM di Perugia, Via Elce di Sotto 8, 06123 Perugia, Italy
3 Consortium for Computational and Materials Sciences (CMS)[2], Via Elce di Sotto, 8, 06123 Perugia, Italy
4 Laboratoire de Chimie et Physique Quantiques, IRSAMC, Université de Toulouse III-Paul Sabatier, 118 Route de Narbonne, 31062 Toulouse, CEDEX 09, France
5 Instituto de Física Fundamental, Consejo Superior de Investigaciones Científicas (IFF-CSIC), Serrano 123, 28006 Madrid, Spain
* Correspondence: yusuf.bramastya@gmail.com or yusuf.apriliyanto@idu.ac.id (Y.B.A.); noelia.faginaslago@unipg.it (N.F.-L.); andrea.lombardi@unipg.it (A.L.)

Supporting Information

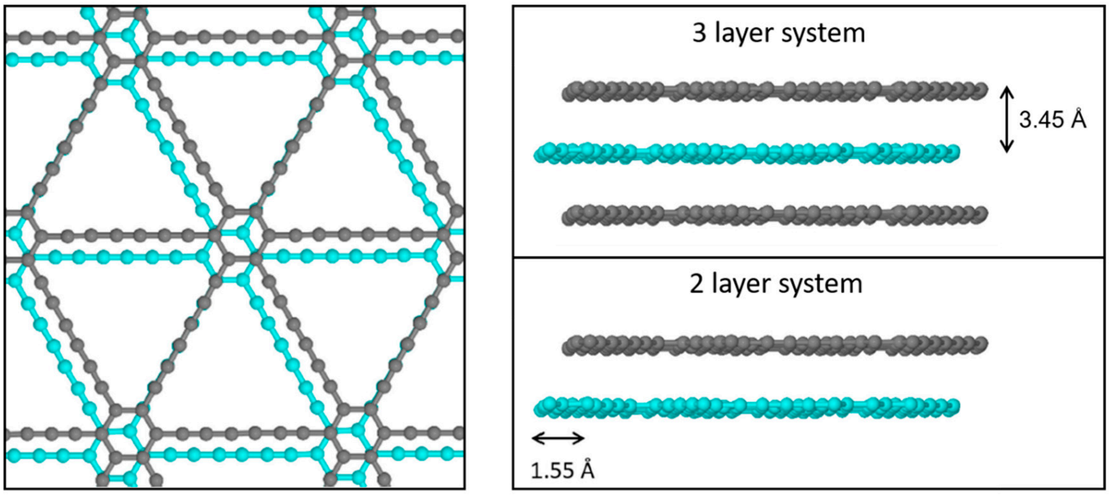

Figure S1. Structural details of multilayer graphtriyne: top view (left panel), side view (right panel).

Table S1. The amount of gas molecules and the corresponding pressure in the simulation box of $CO_2/N_2/H_2O$ gaseous mixtures at 333 K.

Pressure (atm)	CO_2 (molecules)	N_2 (molecules)	H_2O (molecules)
1.00	9	9	9
1.80	17	17	17
2.54	24	24	24
3.18	30	30	30
4.00	38	38	38
4.62	44	44	44
5.47	52	52	52

Table S2. The amount of gas molecules and the corresponding pressure in the simulation box of $CO_2/N_2/H_2O$ gaseous mixtures at 353 K.

Pressure (atm)	CO_2 (molecules)	N_2 (molecules)	H_2O (molecules)
1.00	9	9	9
1.80	16	16	16
2.54	22	22	22
3.18	28	28	28
4.00	36	36	36
4.62	41	41	41
5.47	49	49	49

Table S3. The amount of gas molecules and the corresponding pressure in the simulation box of $CO_2/N_2/H_2O$ gaseous mixtures at 373 K.

Pressure (atm)	CO_2 (molecules)	N_2 (molecules)	H_2O (molecules)
1.00	8	8	8
1.80	15	15	15
2.54	21	21	21
3.18	27	27	27
4.00	34	34	34
4.62	39	39	39
5.47	46	46	46

Table S4. The amount of gas molecules and the corresponding pressure in the simulation box of $CO_2/N_2/H_2O$ gaseous mixtures at 400 K.

Pressure (atm)	CO_2 (molecules)	N_2 (molecules)	H_2O (molecules)
1.00	8	8	8
1.80	14	14	14
2.54	20	20	20
3.18	25	25	25
4.00	31	31	31
4.62	36	36	36
5.47	43	43	43

Table S5. Average of gas permeance of $CO_2/N_2/H_2O$ gaseous mixtures in single layer system.

Temperature (K)	CO_2 (× 10^7 GPU)	N_2 (× 10^7 GPU)	H_2O (× 10^7 GPU)
333	1.600 ± 0.573	0.312 ± 0.005	2.697 ± 0.414
353	1.140 ± 0.198	0.304 ± 0.006	2.324 ± 0.628
373	0.948 ± 0.086	0.232 ± 0.005	1.863 ± 0.181
400	0.707 ± 0.177	0.212 ± 0.003	1.567 ± 0.222

Table S6. Average of gas permeance of $CO_2/N_2/H_2O$ gaseous mixtures in bilayer system.

Temperature (K)	CO_2 (× 10^7 GPU)	N_2 (× 10^7 GPU)	H_2O (× 10^7 GPU)
333	3.000 ± 0.723	0.458 ± 0.059	2.640 ± 0.506
353	2.057 ± 0.585	0.376 ± 0.090	2.115 ± 0.287
373	1.624 ± 0.361	0.350 ± 0.076	1.835 ± 0.386
400	1.390 ± 0.664	0.292 ± 0.077	1.487 ± 0.377

Table S7. Average of gas permeance of $CO_2/N_2/H_2O$ gaseous mixtures in trilayer system.

Temperature (K)	CO_2 (× 10^7 GPU)	N_2 (× 10^7 GPU)	H_2O (× 10^7 GPU)
333	1.563 ± 0.232	0.611 ± 0.164	1.989 ± 0.155
353	1.358 ± 0.199	0.546 ± 0.097	2.008 ± 0.219
373	1.107 ± 0.233	0.480 ± 0.076	1.803 ± 0.106
400	0.977 ± 0.079	0.347 ± 0.071	1.612 ± 0.138

Table S8. Permeance selectivity of $CO_2/N_2/H_2O$ gaseous mixtures in single layer system.

Temperature (K)	CO_2/N_2	H_2O/N_2	H_2O/CO_2
333	5.125 ± 2.640	8.649 ± 2.686	1.688 ± 0.864
353	3.731 ± 1.328	7.634 ± 3.451	2.046 ± 0.909
373	4.078 ± 1.315	8.017 ± 2.640	1.966 ± 0.368
400	3.332 ± 1.321	7.386 ± 2.125	2.217 ± 0.870

Table S9. Permeance selectivity of $CO_2/N_2/H_2O$ gaseous mixtures in bilayer system.

Temperature (K)	CO_2/N_2	H_2O/N_2	H_2O/CO_2
333	6.548 ± 2.421	5.760 ± 1.846	0.880 ± 0.381
353	5.478 ± 2.876	5.632 ± 2.120	1.028 ± 0.432
373	4.636 ± 2.036	5.239 ± 2.238	1.130 ± 0.489
400	4.755 ± 3.517	5.087 ± 2.621	1.070 ± 0.782

Table S10. Permeance selectivity of $CO_2/N_2/H_2O$ gaseous mixtures in trilayer system.

Temperature (K)	CO_2/N_2	H_2O/N_2	H_2O/CO_2
333	2.558 ± 1.066	3.256 ± 1.129	1.273 ± 0.288
353	2.488 ± 0.808	3.680 ± 1.057	1.479 ± 0.377
373	2.305 ± 0.850	3.755 ± 0.813	1.629 ± 0.439
400	2.817 ± 0.805	4.648 ± 1.351	1.650 ± 0.275

Table S11. Total gas uptake (molecules) at 333 K with different pressures.

Initial Pressure (atm)	Single layer			Bilayer			Trilayer		
	CO_2	N_2	H_2O	CO_2	N_2	H_2O	CO_2	N_2	H_2O
1.00	3.278	0.929	1.972	5.204	2.571	4.912	8.144	5.003	7.412
1.80	4.494	1.927	3.179	7.054	2.278	5.187	11.927	3.548	7.600
2.54	3.680	2.370	2.976	9.780	3.081	6.269	13.449	3.432	9.221
3.18	4.481	2.924	3.173	10.574	3.872	5.904	17.193	5.782	10.864
4.00	5.981	3.435	4.595	13.083	4.273	7.990	16.979	5.295	11.398
4.62	7.871	3.897	5.526	12.885	4.961	8.424	18.795	5.891	11.297
5.47	9.297	4.502	6.042	13.920	5.818	10.941	24.977	6.990	16.175

Table S12. Total gas uptake (molecules) at 353 K with different pressures.

Initial Pressure (atm)	Single layer			Bilayer			Trilayer		
	CO_2	N_2	H_2O	CO_2	N_2	H_2O	CO_2	N_2	H_2O
1.00	3.440	1.103	2.039	3.685	1.605	3.123	7.697	2.097	3.130
1.80	2.642	1.833	2.461	5.918	1.947	3.037	10.108	2.598	7.284
2.54	4.655	2.184	2.596	6.807	2.410	4.479	11.737	3.384	7.982
3.18	4.399	2.649	2.968	9.921	3.242	5.950	12.253	3.953	7.425
4.00	4.994	3.241	3.570	8.114	3.398	5.906	12.660	3.801	8.558
4.62	5.741	3.527	4.133	9.612	4.082	7.200	16.170	5.616	11.891
5.47	6.981	4.368	5.355	11.606	5.223	9.383	18.810	6.014	12.820

Table S13. Total gas uptake (molecules) at 373 K with different pressures.

Initial Pressure (atm)	Single layer			Bilayer			Trilayer		
	CO_2	N_2	H_2O	CO_2	N_2	H_2O	CO_2	N_2	H_2O
1.00	2.206	1.135	1.488	4.430	1.550	1.308	3.881	2.406	4.286
1.80	1.995	1.513	1.440	4.085	1.644	4.164	5.769	2.648	5.861
2.54	2.903	1.509	2.262	6.314	2.365	4.342	8.527	2.986	5.746
3.18	3.781	1.734	2.814	6.610	2.927	3.977	10.476	2.852	6.704
4.00	4.904	2.683	3.158	7.064	3.098	5.764	12.857	4.335	8.766
4.62	5.592	3.038	4.336	9.677	4.363	6.507	13.480	4.778	9.640
5.47	6.065	3.764	4.353	9.327	4.536	7.714	11.731	4.860	9.837

Table S14. Total gas uptake (molecules) at 400 K with different pressures.

Initial Pressure (atm)	Single layer			Bilayer			Trilayer		
	CO_2	N_2	H_2O	CO_2	N_2	H_2O	CO_2	N_2	H_2O
1.00	1.683	0.729	0.762	2.389	2.260	3.388	5.033	1.952	2.595
1.80	2.072	1.011	1.648	5.011	1.729	3.080	7.756	2.069	5.512
2.54	2.572	1.593	1.563	4.392	1.654	2.589	8.220	1.642	4.876
3.18	3.089	1.675	2.265	4.959	2.467	3.674	8.177	2.932	5.701
4.00	4.060	2.364	2.908	6.743	2.911	4.768	9.073	3.247	6.219
4.62	3.969	2.701	3.122	7.396	3.321	4.858	10.153	4.231	6.410
5.47	5.503	3.433	3.840	7.411	3.484	5.233	11.411	3.994	7.549

Table S15. Adsorption coefficient (mmol g^{-1} atm^{-1}) of CO_2, N_2, and H_2O in single layer system.

**Temperature (K)**	**CO_2**	**N_2**	**H_2O**
333	0.127 ± 0.030	0.074 ± 0.003	0.087 ± 0.011
353	0.081 ± 0.015	0.067 ± 0.003	0.067 ± 0.009
373	0.097 ± 0.010	0.057 ± 0.007	0.071 ± 0.008
400	0.080 ± 0.008	0.058 ± 0.004	0.064 ± 0.005

Table S16. Adsorption coefficient (mmol g^{-1} atm^{-1}) of CO_2, N_2, and H_2O in bilayer system.

**Temperature (K)**	**CO_2**	**N_2**	**H_2O**
333	0.097 ± 0.011	0.038 ± 0.004	0.062 ± 0.009
353	0.076 ± 0.014	0.038 ± 0.003	0.067 ± 0.008
373	0.062 ± 0.010	0.035 ± 0.004	0.060 ± 0.009
400	0.052 ± 0.009	0.019 ± 0.005	0.026 ± 0.007

Table S17. Adsorption coefficient (mmol g^{-1} atm^{-1}) of CO_2, N_2, and H_2O in trilayer system.

**Temperature (K)**	**CO_2**	**N_2**	**H_2O**
333	0.106 ± 0.012	0.019 ± 0.008	0.056 ± 0.010
353	0.073 ± 0.008	0.028 ± 0.004	0.061 ± 0.010
373	0.067 ± 0.013	0.020 ± 0.003	0.043 ± 0.005
400	0.039 ± 0.005	0.019 ± 0.004	0.028 ± 0.006

Table S18. Interlayer adsorption selectivity of $CO_2/N_2/H_2O$ gaseous mixture.

Temperature (K)	Gas	System	Interlayer adsorption selectivity						
			**1.00 atm**	**1.80 atm**	**2.54 atm**	**3.18 atm**	**4.00 atm**	**4.62 atm**	**5.47 atm**
333	CO_2/N_2	Bilayer	3.01	15.06	13.54	10.90	20.23	11.16	6.95
		Trilayer	7.16	13.50	15.96	10.05	11.86	11.12	13.85
	H_2O/N_2	Bilayer	2.85	10.35	8.37	4.85	10.93	6.97	6.76
		Trilayer	3.86	4.99	9.20	4.52	6.76	5.40	7.28
	CO_2/H_2O	Bilayer	1.06	1.46	1.62	2.25	1.85	1.60	1.03
		Trilayer	1.86	2.71	1.73	2.22	1.75	2.06	1.90
353	CO_2/N_2	Bilayer	8.34	18.31	10.28	12.70	13.51	7.61	6.13
		Trilayer	22.48	24.51	13.80	12.67	12.58	10.17	11.73
	H_2O/N_2	Bilayer	7.60	5.87	6.44	5.97	10.34	7.00	6.15
		Trilayer	2.82	11.94	7.45	5.63	8.12	6.45	6.81
	CO_2/H_2O	Bilayer	1.10	3.12	1.60	2.13	1.31	1.09	1.00
		Trilayer	7.97	2.05	1.85	2.25	1.55	1.58	1.72
373	CO_2/N_2	Bilayer	6.20	13.52	9.53	6.10	10.93	8.43	8.19
		Trilayer	2.35	4.80	6.91	18.15	10.84	8.96	9.57
	H_2O/N_2	Bilayer	0.83	14.51	6.22	2.69	13.62	6.19	7.08
		Trilayer	3.37	4.95	4.30	9.03	5.99	5.78	8.69
	CO_2/H_2O	Bilayer	7.45	0.93	1.53	2.27	0.80	1.36	1.16
		Trilayer	0.70	0.97	1.61	2.01	1.81	1.55	1.10
400	CO_2/N_2	Bilayer	1.72	6.89	14.73	7.13	12.33	8.82	7.12
		Trilayer	6.27	11.31	42.45	5.80	9.77	7.20	11.20
	H_2O/N_2	Bilayer	2.86	3.39	8.91	5.13	9.27	6.83	6.72
		Trilayer	1.65	5.89	17.86	3.73	6.05	3.90	6.48
	CO_2/H_2O	Bilayer	0.60	2.03	1.65	1.39	1.33	1.29	1.06
		Trilayer	3.80	1.92	2.38	1.55	1.62	1.85	1.73
